# 3D Facial Analysis in Acromegaly: Gender-Specific Features and Clinical Correlations

**DOI:** 10.3389/fendo.2018.00722

**Published:** 2018-11-29

**Authors:** Xiaopeng Guo, Tian Meng, Jiuzuo Huang, Xiaojun Wang, Wei Lian, Kan Deng, Lu Gao, Zihao Wang, Bing Xing, Xiao Long

**Affiliations:** ^1^Department of Neurosurgery, Peking Union Medical College Hospital, Chinese Academy of Medical Sciences & Peking Union Medical College, Beijing, China; ^2^China Pituitary Disease Registry Center, Beijing, China; ^3^China Pituitary Adenoma Specialist Council, Beijing, China; ^4^Department of Plastic Surgery, Peking Union Medical College Hospital, Chinese Academy of Medical Sciences & Peking Union Medical College, Beijing, China

**Keywords:** three-dimensional stereophotography, acromegaly, facial changes, gender, insulin-like growth factor 1

## Abstract

**Objective:** Quantitative investigations of facial changes in acromegaly are rare. A new imaging technique, three-dimensional (3D) stereophotography, can accurately quantify whole facial changes. We aimed to measure facial characteristics in acromegaly patients using 3D stereophotography, analyze gender-specific features, and explore clinical influencing factors.

**Design:** Single-center case-control study.

**Methods:** Thirty-nine acromegaly patients and 39 age- and gender-matched healthy subjects were prospectively enrolled. 3D stereophotography was performed, and facial lines and angles were quantified for each subject. Clinical information for each acromegaly patient was acquired.

**Results:** The nose width, length, height and depth were longer; the upper and lower lips were thicker; the face length, face width and gonion-gnathion distances were longer; and the nasofrontal and columella-labial angles were smaller in the acromegaly patients, especially in males, than in the healthy controls, with statistical significance (*p* < 0.05). No differences were found in the face breadth, columella-labial angle, or nose length, height or depth between the female patient and healthy control groups. The insulin-like growth factor 1 (IGF-1) levels in the acromegaly patients were linearly and positively correlated with the nose width (*p* = 0.006) and gonion-gnathion distance (*p* = 0.029) and linearly and negatively correlated with the nasofrontal angle (*p* = 0.026).

**Conclusions:** The acromegaly patients' facial changes exhibit a unique trend, and the characteristics are not identical between genders. 3D stereophotography is an accurate and reliable tool for investigating facial characteristics. Recognizing the above facial features might be potential to assist in the early diagnosis and timely treatment of acromegaly and aid in predicting the severity of systemic complications.

## Introduction

High levels of growth hormone (GH) and insulin-like growth factor 1 (IGF-1) in acromegaly patients, which are mostly due to a GH-secreting pituitary adenoma, contribute to tissue proliferation and hypertrophy throughout the body (1, 2). Abnormal facial bone hyperosteogeny and facial soft tissue hypertrophy chronically deform the faces of acromegaly patients, leading to a lack of self-confidence and psychological trauma while decreasing quality of life (1, 3, 4–6). The facial changes of acromegaly patients are relatively insidious, and the average disease duration at diagnosis is approximately 10 years (7). At the time of the endocrine diagnosis, the cavernous sinus is always aggravated by the pituitary tumor, and patients tend to be diagnosed with systemic complications, such as cardiovascular disease, resulting in poor therapeutic effects and an unsatisfactory long-term prognosis. Quantitative analysis of facial changes is necessary for the accurate identification of the acromegaly-specific face, which also assists in early facial recognition and in shortening the exposure duration to excessive GH and IGF-1.

Two-dimensional (2D) photography is commonly used in acromegalic facial analysis. Several previous studies collected 2D facial photographs and used computer software to distinguish acromegaly patients from a healthy population, with so-called artificial intelligence (8–12). In recent years, three-dimensional (3D) stereophotography has become increasingly popular for image reconstruction, in which the lines and angles are generally more accurately measured than in the 2D photograph (only measures the profile on the mid-sagittal plane of the face) (13–15). Studies on the use of 3D stereophotography for acromegaly patients are scarce. For the first time, (16) applied the 3D stereophotograph to the facial analysis of post-treatment acromegaly patients. In addition, 3D stereophotography could also be used in the evaluation of the acromegalic extremities (17).

Until now, an enlarged nose and face, a protruding mandible and forehead and thickened lips have been the facial characteristics that are superficially associated with acromegaly patients (6). However, to the best of our knowledge, there have been no studies using 3D images to assess the facial changes of pre-treatment active acromegaly patients, even though 3D images may provide more information in the field of facial recognition for acromegaly patients. In addition, most doctors have the clinical experience that the facial changes of male acromegaly patients can be more easily recognized than those of female patients, but whether the degree of quantitative changes between the genders is identical is unclear. Moreover, in our previous study series on acromegaly, we found that the level of IGF-1 is significantly correlated with posterior pharyngeal wall thickness, the development of difficulty with intubation and choroidal thickness, and the disease duration is significantly related to the choroidal thickness and expansion of great vessels (18–21). Thus, in this study, we hypothesized that hormone levels and disease duration also correlate with the facial changes.

Therefore, we used 3D stereophotography to record, reconstruct and measure the faces of acromegaly patients and age- and gender-matched healthy controls in one of the largest pituitary tumor centers in China. The aim of this study was to quantitatively analyze the facial characteristics and facial changes in acromegaly before treatment, identify the differences and features between the genders, and explore the correlations between facial changes and the GH level, the IGF-1 level and disease duration.

## Subjects and methods

### Study population

Acromegaly patients admitted to the Department of Neurosurgery of Peking Union Medical College Hospital from January 2017 to April 2018 were enrolled in this study. The inclusion criteria were as follows: (1) typical presentations of acromegaly (6); (2) GH and IGF-1 levels in accordance with the endocrine diagnostic criteria (2); (3) a finding of a pituitary adenoma on contrast-enhanced MRI; (4) adult patients (>18 years) with no gender restriction; and (5) other pituitary related hormones, including prolactin, follicle-stimulating hormone, luteinizing hormone, adrenocorticotropic hormone, cortisol, and thyroid stimulating hormone, within the normal range to avoid potential effects on the facial structures. Age- and gender-matched healthy people were enrolled in the control group via advertisements. The age gap between acromegaly patients and matched healthy subjects did not exceed 3 years.

This study was approved by the Institutional Review Board at Peking Union Medical College Hospital, Chinese Academy of Medical Sciences (Ethical Number: ZS-1324). Each patient signed an informed consent document before enrollment.

### Study design

This was a prospectively conducted, single-center, case-control study. The age (year), height (cm), weight (kg) and body mass index (BMI) (kg/m^2^) of each group were recorded. We also collected data on the durations of disease (months) and levels of hormones, including GH (ng/ml), IGF-1 (ng/ml) and the GH nadir (ng/ml), of the acromegaly patients. The disease duration was defined as the period from the onset of acromegalic presentations to the time of the endocrine diagnosis. Blood was collected at 6 a.m. in the morning after 8h fasting period. GH and IGF-1 were measured using a chemiluminescence assay and an IMMULITE 2000 analyzer in clinical laboratory of Peking Union Medical College Hospital. Methods for hormone measurement and normal values could refer to our previous studies (18, 19). GH nadir was achieved after oral glucose tolerance test.

All subjects underwent 3D stereophotography after enrollment. A qualified doctor (MT) with over 5 years of experience conducted the photography, and the photos were post-processed with the VECTRA software (Canfield Scientific, Inc. USA).

### Three-dimensional stereophotography

The 3D stereophotography was performed using the VECTRA H1-270 handheld camera (Canfield Scientific, Inc. USA), and facial images were captured at 3 different angles for each subject. The patients were required to sit in a chair, stare ahead and remain completely still throughout the 3 image captures. The camera was first positioned in front of the subject, leveling with the nose. After aiming and converging the green dots between the nose and upper lip, the first photograph was taken. Then, the camera was positioned at a 45°C angle from the front to either the lateral side of the face, leveling with the chest. After aiming and converging the green dots at the middle of the cheek, the second and third photographs were taken.

**Figure 1:**
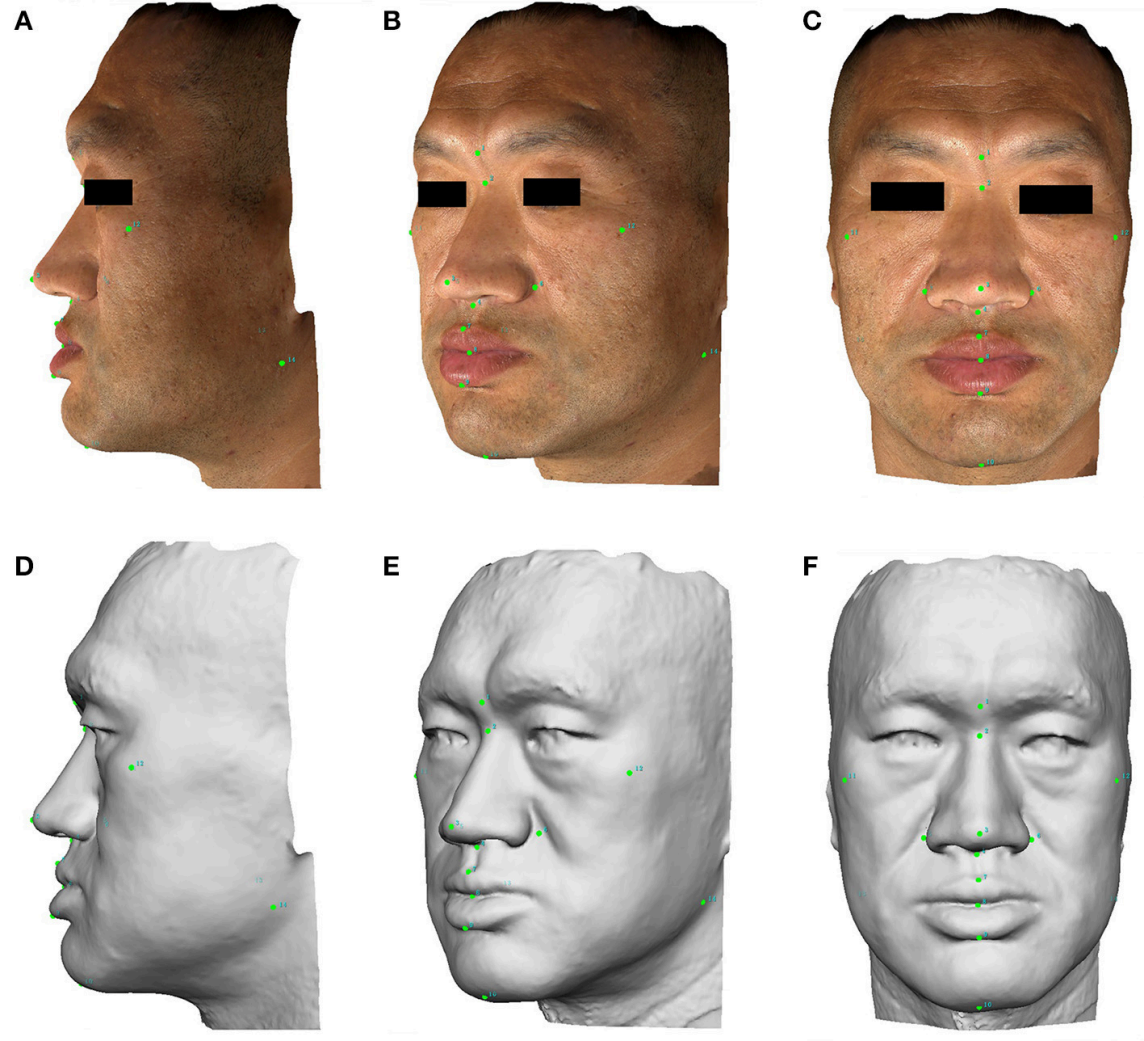
The lateral, anterolateral, and anterior images of facial 3D photography for a male acromegaly patient. Points are labeled on the actual 3D images **(A–C)** and gray-mode 3D images **(D–F)** for the measurement of facial lines and angles. A written informed consent was obtained from the individual for the publication of this image.

Three facial images were captured for each subject and then automatically stitched into one 3D image. A total of 14 points were labeled on the 3D image. The points were labeled numerically as follows: (1) glabella; (2) nasion; (3) pronasale; (4) subnasale; (5) right alare; (6) left alare; (7) labiale superius; (8) stomion; (9) labiale inferius; (10) gnathion; (11) right zygion; (12) left zygion; (13) right gonion; and (14) left gonion. A gray-mode 3D image was transformed from the actual color 3D image to reduce color distraction for measurement and calculation (Figure 1).

The facial lines and angles for measurement were divided into three groups, namely, the nose-related, lip-related and facial contour-related parameter groups. The 7 nose-related parameters included (a) nose length (2–3); (b) nose depth (3–4); (c) nose height (2–4); (d) nose width (5–6); (e) nasal width index [(5–6)/(2–4)]; (f) nasofrontal angle (1–2–3); and (g) columella-labial angle (3–4–7). The 3 lip-related parameters included (a) upper vermilion height (7–8); (b) lower vermilion height (8–9); and (c) vermilion height (7–9). The 4 facial contour-related parameters included (a) morphological face length (2–10); (b) face breadth (11–12); (c) facial length index [(11–12)/(2–10)]; and (d) gonion-gnathion distance (10–13/14).

### Statistical analysis

The statistical analysis of the results was conducted with SPSS (SPSS Inc., version 17.0, USA) and GraphPad Prism (GraphPad Inc., version 5, USA). The data are shown as the means ± standard deviations, numbers or percentages. Levene's test was used to evaluate the distribution of quantitative data, and Student's *t*-test was used for data comparisons. The *r*^2^ value revealed the fitness of the linear regression with the actual results. Statistical significance was defined as *p* < 0.05.

## Results

### Study population

In this study, 39 acromegaly patients and 39 healthy controls were enrolled (23 males and 16 females). The general information of the subjects is presented in Table 1. For the acromegaly patients, the average disease duration was 72 months, and the average GH level, GH nadir level and IGF-1 level were 30.8, 22.8, and 875.1 ng/ml, respectively.

**Table 1:** General information of the subjects.

	**Acromegaly patients**			**Healthy controls**		
	**Total (*n* = 39)**	**Male (*n* = 23)**	**Female (*n* = 16)**	**Total (*n* = 39)**	**Male (*n* = 23)**	**Female (*n* = 16)**
Age (year)	42.9 ± 10.86	39.4 ± 8.03	47.9 ± 12.60	43.3 ± 11.00	39.9 ± 9.75	48.2 ± 11.11
Height (cm)	171.7 ± 8.31	177.1 ± 5.36	163.9 ± 4.73	170.0 ± 7.59	174.8 ± 5.68	163.1 ± 3.56
Weight (kg)	78.1 ± 15.00**	85.8 ± 13.72**	67.0 ± 8.36	69.4 ± 9.82	74.1 ± 9.74	62.7 ± 4.88
BMI (kg/m^2^)	26.3 ± 3.24**	27.2 ± 3.27**	24.9 ± 2.73	23.9 ± 2.02	24.1 ± 2.36	23.6 ± 1.38
Disease duration (month)	72.0 ± 55.30	73.3 ± 64.06	70.1 ± 41.44	–	–	–
Fasting GH level (ng/ml)	30.8 ± 40.66	29.9 ± 36.30	32.2 ± 47.47	–	–	–
GH Nadir after OGTT (ng/ml)	22.8 ± 29.90	21.6 ± 27.26	24.4 ± 34.21	–	–	–
Fasting IGF-1 level (ng/ml)	875.1 ± 234.39	934.9 ± 213.61	789.3 ± 242.74	–	–	–

*BMI, body mass index; GH, growth hormone; OGTT, oral glucose tolerance test; IGF-1, insulin-like growth factor 1*.

** *means the difference between acromegaly patients and healthy controls was significant (p < 0.01)*.

No differences in the average age or height were found between the acromegaly patients and healthy controls. However, the weight (*p* = 0.004) and BMI (*p* < 0.001) of the acromegaly patients were larger than those of the healthy controls. In male patients, the weight (*p* = 0.002) and BMI (*p* = 0.001) were larger than those the healthy subjects, whereas those of the female patients were not.

### Quantitative facial changes of the acromegaly patients

The facial parameters, including the nose-related, lip-related and facial contour-related parameters, were measured in both groups and are presented in Table 2.

**Table 2:** Quantitative facial changes of acromegaly patients compared with healthy controls.

	**Acromegaly patients (*****n*** = **39)**		**Healthy controls (*****n*** = **39)**		***P*-value**	**Trends**
	**Means ± SD**	**Range**	**Means ± SD**	**Range**	
**NOSE-RELATED INDEXES**						
Nose length (mm)	47.3 ± 6.36	33.8–60.9	43.8 ± 3.46	35.7–50.3	0.004	Up
Nose depth (mm)	20.5 ± 2.48	15.4–25.2	18.9 ± 1.85	14.5–23.8	0.002	Up
Nose height (mm)	54.4 ± 5.34	40.2–65.9	51.7 ± 3.36	43.6–58.8	0.012	Up
Nose width (mm)	48.9 ± 4.44	41.6–61.7	42.0 ± 2.80	36.1–47.4	<0.0001	Up
Nasal width index (%)	90.4 ± 8.76	74.6–119.4	81.4 ± 6.77	69.4–95.1	<0.0001	Up
Nasofrontal angle (°)	136.4 ± 9.01	116.0–150.0	145.7 ± 6.38	129.0–157.0	<0.0001	Down
Columella-labial angle (°)	94.7 ± 12.0	64.0–119.0	100.3 ± 7.69	84.0–113.0	0.018	Down
**LIP-RELATED INDEXES**						
Upper vermilion height (mm)	11.5 ± 1.93	6.8–16.4	9.0 ± 1.72	5.4–13.8	<0.0001	Up
Lower vermilion height (mm)	12.7 ± 2.33	8.3–17.8	8.6 ± 1.54	4.4–11.1	<0.0001	Up
Vermilion height (mm)	24.1 ± 3.60	17.5–31.6	17.6 ± 2.91	9.9–23.8	<0.0001	Up
**BONE-RELATED INDEXES**						
Morphological face length (mm)	130.8 ± 9.76	113.4–153.2	118.7 ± 7.74	105.2–137.1	<0.0001	Up
Face breadth (mm)	138.7 ± 10.81	117.9–157.3	126.7 ± 7.26	113.2–143.5	<0.0001	Up
Facial length index (%)	94.6 ± 6.51	77.5–108.8	93.9 ± 6.61	82.5–110.7	0.633	Level
Gonion-gnathion distance (mm)	114.7 ± 8.22	94.3–132.0	108.7 ± 8.09	93.1–128.6	0.002	Up

*P value indicates the difference between acromegaly patients and healthy controls; p < 0.05 means the difference is significant*.

#### Nose-related parameters

Compared with the healthy controls, the nose length (average change = 3.5 mm, *p* = 0.004), nose height (average change = 2.7 mm, *p* = 0.012), nose depth (average change = 1.6 mm, *p* = 0.002) and nose width (average change = 6.9 mm, *p* < 0.001) were remarkably larger in the acromegaly patients. Regarding the manner of nose development, the nasal width index was significantly larger in the acromegaly patients (average change = 9.0%, *p* < 0.001). Both the nasofrontal angle (average change = 9.3°C, *p* < 0.001) and the columella-labial angle (average change = 5.6°, *p* = 0.018) were decreased in the acromegaly patients.

#### Lip-related parameters

The height of the vermilion was larger in the acromegaly patients (average change = 6.5 mm, *p* < 0.001). The two parts of the vermilion height, namely, the upper vermilion height (average change = 2.5 mm, *p* < 0.001) and the lower vermilion height (average change = 4.1 mm, *p* < 0.001), were larger in the acromegaly patients than in the healthy controls.

#### Facial contour-related parameters

The morphological face length (average change = 12.1 mm, *p* < 0.001) and face breadth (average change = 12.0 mm, *p* < 0.001) were both elevated in the acromegaly patients, whereas the manner of facial development, namely, the facial length index, was not significantly different from that of the healthy controls (94.6 ± 6.51% vs. 93.9 ± 6.61%, *p* = 0.663). The gonion-gnathion distance was also larger in the acromegaly patients (average change = 6.0 mm, *p* = 0.002).

### Gender-specific facial features in acromegaly

**Figure 2:**
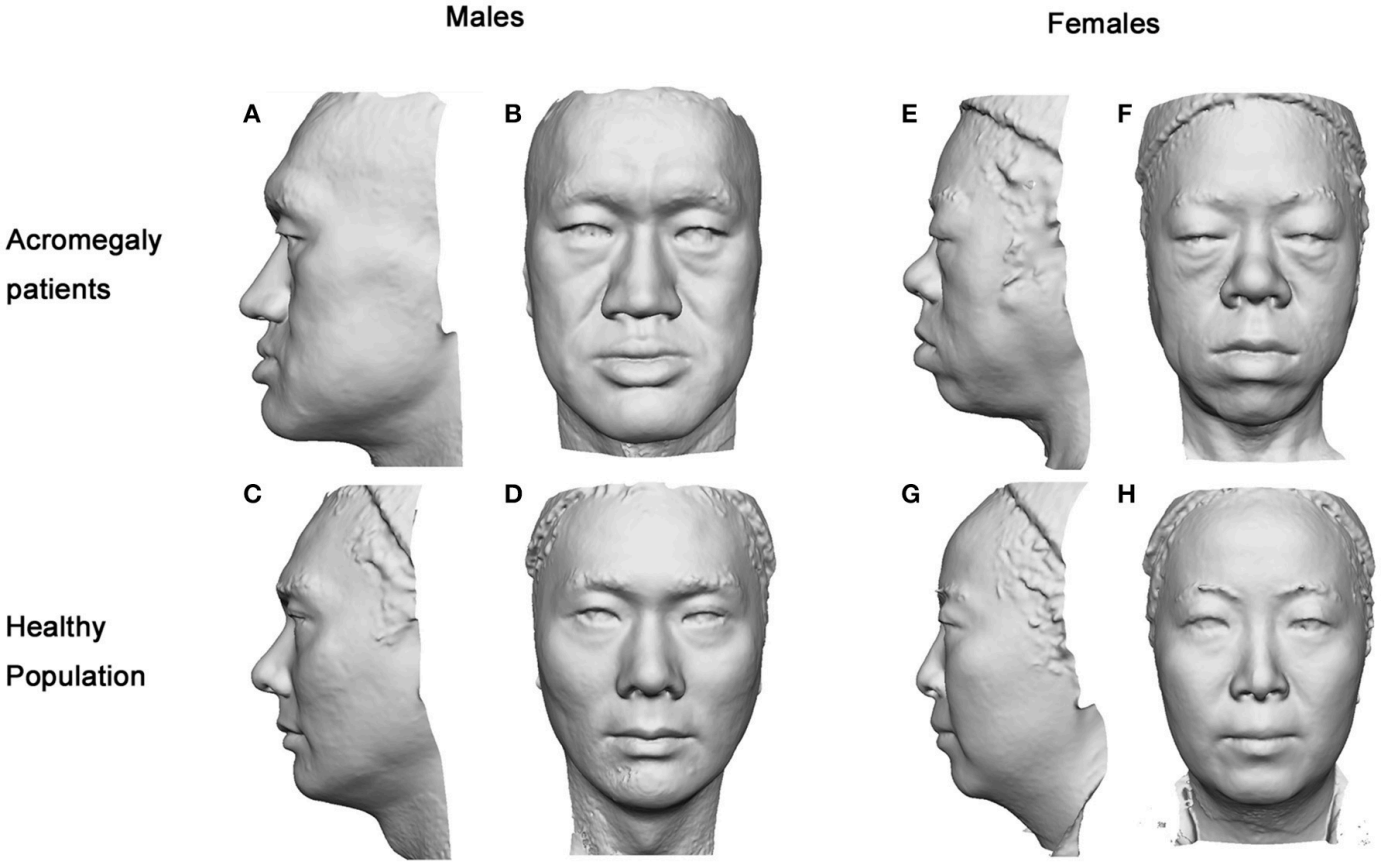
Gray-mode 3D images of the acromegaly patients and healthy controls. The facial characteristics of the acromegaly patients were remarkable as presented in either gender. In male patients **(A,B)**, the nasofrontal angle and columella-labial angle were smaller, the gonion-gnathion distance was longer, the face contour and nose were larger, and the vermilions were thicker than those of the healthy males **(C,D)**. In female patients **(E,F)**, the face contour was more oval, the nose was wider, the nasofrontal angle was smaller, the gonion-gnathion distance was longer, and the vermilions were thicker than those of the healthy females **(G,H)**.

The facial parameters of the two groups were compared separately for each gender. Typical 3D photography of both groups is shown in Figure 2. We found that the facial changes of the male patients were in line with the general trends of the acromegaly group and were more typical than those of the female patients. In the male patients, the nose length, nose height, nose depth, nose width and nasal width index were increased, the nasofrontal angle and columella-labial angle were decreased, the upper and lower vermilions were thickened, and the morphological face length, face breadth and gonion-gnathion distance were increased compared with those of the healthy males (details in Table 3).

**Table 3:** Gender-specific facial features in acromegaly.

	**Males**			**Females**		
	**Acromegaly (*n* = 23)**	**Control (*n* = 23)**	***P1* value**	**Acromegaly (*n* = 16)**	**Control (*n* = 16)**	***P2* value**
**NOSE-RELATED INDEXES**						
Nose length (mm)	51.0 ± 4.75	45.4 ± 3.22	<0.001	42.1 ± 4.41	41.7 ± 2.57	0.752
Nasal depth (mm)	21.2 ± 2.40	19.1 ± 1.87	0.002	19.6 ± 2.33	18.6 ± 1.82	0.202
Nose height (mm)	57.2 ± 3.77	52.9 ± 3.58	<0.001	50.3 ± 4.68	50.1 ± 2.22	0.850
Nose width (mm)	51.2 ± 4.14	43.2 ± 2.30	<0.001	45.6 ± 2.25	40.2 ± 2.50	<0.001
Nasal width index (%)	89.8 ± 7.30	82.1 ± 6.91	0.001	91.4 ± 10.70	80.5 ± 6.66	0.002
Nasofrontal angle (°)	132.7 ± 9.10	144.0 ± 6.74	<0.001	141.8 ± 5.68	148.1 ± 5.12	0.002
Columella-labial angle (°)	90.1 ± 11.91	97.9 ± 7.85	0.012	101.4 ± 8.60	103.6 ± 6.23	0.416
**LIP-RELATED INDEXES**						
Upper vermilion height (mm)	11.8 ± 2.30	9.5 ± 1.97	0.001	10.9 ± 1.09	8.3 ± 0.90	<0.001
Lower vermilion height (mm)	13.5 ± 2.29	8.9 ± 1.81	<0.001	11.5 ± 1.91	8.3 ± 0.99	<0.001
Vermilion height (mm)	25.3 ± 3.84	18.3 ± 3.41	<0.001	22.5 ± 2.46	16.5 ± 1.54	<0.001
**BONE-RELATED INDEXES**						
Morphological face length (mm)	136.6 ± 7.26	122.2 ± 7.80	<0.001	122.5 ± 6.34	113.8 ± 4.30	<0.001
Face breadth (mm)	144.5 ± 7.07	127.7 ± 7.80	<0.001	130.3 ± 9.77	125.4 ± 6.41	0.102
Facial length index (%)	94.6 ± 4.83	95.9 ± 6.44	0.454	94.5 ± 8.55	91.0 ± 5.89	0.180
Gonion-gnathion distance (mm)	117.8 ± 7.90	112.0 ± 7.24	0.012	110.3 ± 6.60	103.9 ± 6.89	0.012

*P1 indicates the difference between male patients and healthy males; P2 indicates the difference between female patients and healthy females; p < 0.05 means the difference is significant*.

In the female acromegaly patients, some of the facial changes were not as remarkable. Compared with the healthy females, the nose width (*p* < 0.001) and nasal width index (*p* = 0.002) were increased, the nasofrontal angle (*p* = 0.002) was decreased, the upper and lower lips were thickened (*p* < 0.001), and the gonion-gnathion distance (*p* = 0.012) and morphological face length (*p* < 0.001) were increased. However, no differences were found in terms of the nose length (*p* = 0.752), nose height (*p* = 0.202), nose depth (*p* = 0.850), columella-labial angle (*p* = 0.416) or face breadth (*p* = 0.102) between the female patients and healthy female controls.

### Related risk factors for acromegalic facial changes

**Figure 3:**
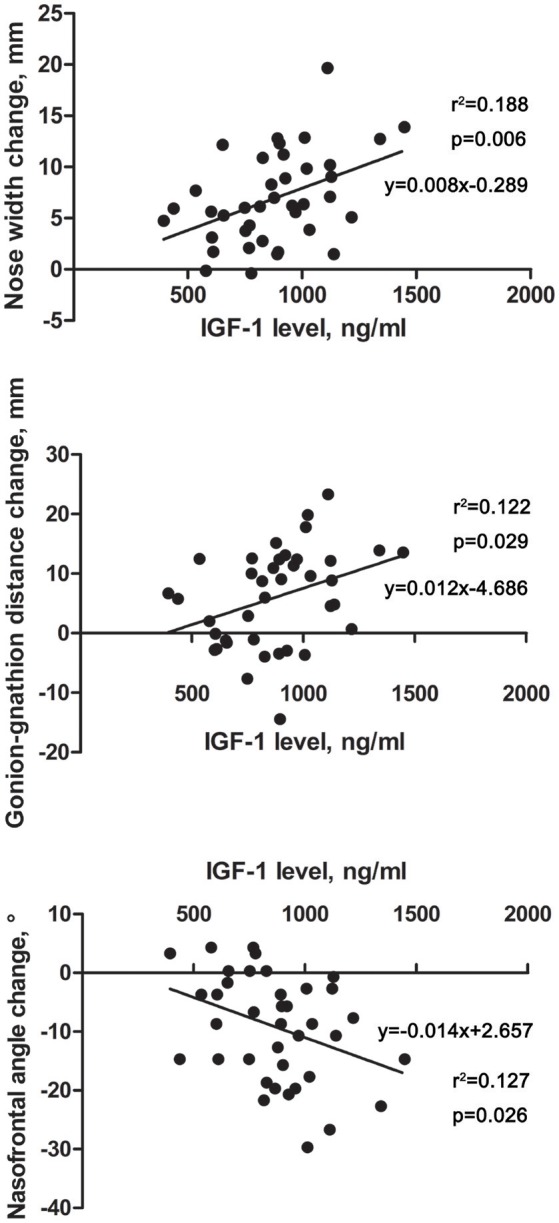
Scatter diagrams revealing the correlations between IGF-1 level and facial parameter changes in acromegaly patients. The horizontal axis indicates the level of IGF-1, and the vertical axis indicates the facial parameter changes of the acromegaly patients compared with those of the healthy controls. Each point in the scatter diagrams represents the change in one patient. Linear-regression analysis was performed, and the fitting line and formula are labeled.

Linear-regression analysis was performed between the facial changes of the acromegaly patients and the hormone levels and disease duration (Supplmentary Table 1). No correlations were found between the facial changes and the patients' disease duration, GH level or GH nadir level, whereas the IGF-1 level was linearly correlated with several facial changes (Figure 3). The results showed that the IGF-1 levels were positively correlated with a nose width change (*p* = 0.006) and gonion-gnathion distance change (*p* = 0.029) but negatively correlated with a nasofrontal angle change (*p* = 0.026).

## Discussion

This study was the first to use 3D stereophotography to analyze the quantitative facial changes, gender-specific facial characteristics and related clinical factors of active acromegaly. Our results showed that compared with the healthy controls, the acromegaly patients showed varying degrees of quantitatively changes in their nose, lips, mandibles, and facial contours. The acromegalic facial characteristics were gender-specific. The characteristics of the male acromegaly patients were typical and remarkable, whereas some of the quantitative facial parameters of the female patients were not different from those of the healthy female controls. The fasting IGF-1 level, but not the GH level or disease duration, was linearly correlated with the changes in nose width, gonion-gnathion distance and nasofrontal angle.

The facial changes of acromegaly are caused by the chronic effects of GH and IGF-1 on the bone, cartilage and soft tissue (16, 22). The nose and lip changes are more likely the external presentations of cartilage and soft tissue changes, whereas facial contour, mandible and nasofrontal angle changes are more likely the external presentations of bone changes. A previous study involving a cephalometric assessment confirmed the prominence of the nose, the decreased columella-labial angle and the thickened lips in acromegaly (23). However, the 2D photography analysis in the above study only measured the facial profile on the mid-sagittal plane, providing fewer available parameters than the 3D images and possibly adding measuring deviations.

In this study, the nose of the acromegaly patients in all directions seemed larger than that of the healthy controls. Interestingly, the width/height ratio was significantly elevated in acromegaly, indicating that the change in the width of the nose was more typical than the changes in the other nose-related parameters. Although the depth and height were also larger, the nose of the acromegaly patient looked more flat than straight or tall. The nose changes of the female patients, who only presented with a larger nose width, were not as typical as those of the male patients. In terms of acromegalic nose changes, this result explained our clinical impression that female patients were not as easily recognized as male patients.

Lip thickening is obvious in acromegaly. Our results showed that the whole vermilion of the acromegaly patients was thicker than that of the healthy controls, with that of the male patients increasing by 7 mm and that of the female patients increasing by 6 mm. In this study, the upper vermilion height was larger than the lower vermilion height in the healthy controls, whereas the lower vermilion height was larger than the upper vermilion height in the acromegaly patients, suggesting that the growth speeds of the lips (lower > upper) were different in acromegaly. Bavbek et al. (23) demonstrated that the upper lip sulcus was deeper and that the lower lip was protruded more in the acromegaly patients. The co-existence of lower lip ectropion and lip thickening made the 3D image measurement of the lower lip (vermilion) larger than the actual size and led to the phenomenon that the lower vermilion height was larger than the upper one in acromegaly. Whether the receptor distributions for GH and IGF-1 are different in the upper or lower lip will need to be verified in future basic animal research.

The decreased facial angles portrayed a more tridimensional face in the acromegaly patients, which accorded with the conclusion of Bavbek et al. (23). The nasofrontal angle and columella-labial angle are influenced by multiple facial structures, including the forehead, geison, maxilla, nasal septum and lips. Previous studies have shown that the anterior skull base is longer and that the geison and maxilla are more prominent. Combining the findings in this study that the nose is deeper and the upper lip is thicker, the cause of the smaller nasofrontal angle and columella-labial angle can be partially explained. Statistically insignificant changes in the length and depth of the nose and upper lip thickness of the female patients might explain why the columella-labial angle was not decreased in female patients.

In this study, the mandible was elongated in both the male and female patients, and the width and length were proportionally increased; these findings were identical to the results of Wagenmakers et al. (16). In female patients, however, the face width was similar to that of the controls, while the face length was larger than that of the healthy controls. Thus, a longer face might be another typical face contour characteristic of female acromegaly patients. Given that the gonadal hormones of the enrolled acromegaly patients were all within the normal ranges, we hypothesized that the differences in the patients' faces between the genders might be related to an unbalanced hormone receptor distribution.

The early diagnosis of acromegaly is rather difficult. Many acromegaly patients are ultimately diagnosed in the Endocrinology or Neurosurgery department because of the poor therapeutic effects in terms of the nasal septum, cardiomyopathy or sleep apnea hypopnea without a primary diagnosis of acromegaly (6, 7, 24). However, although each change in the facial parameters was slight, a combination of the facial characteristics, including face contour, nose and lips, would benefit early facial recognition in acromegaly, especially in males. A longer face and flat wide nose are typical features of female acromegaly patients. Therefore, we recommend that doctors become familiar with these facial characteristics and try to communicate them to the public. Moreover, future 3D-image-based artificial intelligence will provide a more satisfying perspective and needs to be regarded as more important.

High levels of GH and IGF-1 and a longer disease duration were independent risk factors for several complications in acromegaly patients, including cardiovascular disease, obstructive sleep apnea hypopnea syndrome, body composition changes, thyroid nodules and retinal choroid diseases (6, 18–21). In our study, the IGF-1 level, but not the GH level or disease duration, was linearly correlated with the nose width, mandible length and nasofrontal angle. The underlying mechanisms of the selective effects of IGF-1 on the face need to be addressed. Our previous studies discovered that the IGF-1 level was correlated with the pharyngeal wall thickness and the development of difficulty with intubation. Therefore, facial changes might provide a clue for predicting the degree of airway stenosis and the possibility of difficulty with intubation to some extent.

In summary, this study quantitatively analyzed the characteristics of facial changes in acromegaly patients using 3D stereophotography, and the results might be able to increase the proportion of early facial recognition and diagnosis. The facial characteristics of the male patients were typical, whereas the facial changes of the female patients were not as remarkable as those of the male patients. The level of IGF-1 was correlated with some facial parameters and might have the potential to assist in predicting the severity of acromegalic complications.

## Author contributions

XG, TM, XL, and BX designed the study; XG, JH, XW, WL, KD, LG, and ZW enrolled the patients and recorded clinical information; TM conducted the photography and analyzed the data; XG and TM wrote the manuscript; and XL and BX revised the manuscript.

### Conflict of interest statement

The authors declare that the research was conducted in the absence of any commercial or financial relationships that could be construed as a potential conflict of interest.

## Abbreviations

GH: growth hormone
IGF-1: insulin-like growth factor 1
BMI: body mass index
2D: two-dimensional
3D: three-dimensional.
