# Supplementary material for: 3D Facial Analysis in Acromegaly: Gender-Specific Features and Clinical Correlations
Source: Front Endocrinol (Lausanne). 2018 Nov 29;9:722. doi: 10.3389/fendo.2018.00722 (PMC6281698; doi:10.3389/fendo.2018.00722)
Supplement: Supplementary file 1 [file Table_1.DOCX]

| **Supplementary Table 1 Linear regression results between facial parameter changes and hormone effects.** | | | | | | | | | | | | | |
| --- | --- | --- | --- | --- | --- | --- | --- | --- | --- | --- | --- | --- | --- |
|  |  | **Facial parameter changes (mean, range)** | **Fasting IGF-1** | |  | **Fasting GH** | |  | **GH Nadir** | |  | **Disease duration** | |
|  |  |  | **r^2^** | **P value** |  | **r^2^** | **P value** |  | **r^2^** | **P value** |  | **r^2^** | **P value** |
| Nose-related indexes | |  |  |  |  |  |  |  |  |  |  |  |  |
|  | Nose length (mm) | 3.5, (17.1 to -10.0) | 0.077 | 0.087 |  | 0.045 | 0.195 |  | 0.060 | 0.133 |  | 0.045 | 0.195 |
|  | Nose depth (mm) | 1.6, (6.3 to -3.5) | 0.035 | 0.254 |  | 0.070 | 0.103 |  | 0.017 | 0.435 |  | 0.070 | 0.103 |
|  | Nose height (mm) | 2.7, (14.2 to -11.5) | 0.085 | 0.072 |  | 0.046 | 0.190 |  | 0.041 | 0.217 |  | 0.046 | 0.190 |
|  | Nose width (mm) | 6.9, (19.7 to -0.4) | 0.188 | 0.006 |  | 0.002 | 0.801 |  | 0.002 | 0.780 |  | 0.002 | 0.801 |
|  | Nasal width/height index (%) | 9.0, (38.0 to -6.8) | 0.002 | 0.808 |  | 0.031 | 0.287 |  | 0.031 | 0.287 |  | 0.031 | 0.287 |
|  | Nasofrontal angle (°) | 9.3, (-29.7 to 4.3) | 0.127 | 0.026 |  | 0.008 | 0.578 |  | 0.008 | 0.600 |  | 0.008 | 0.578 |
|  | Columella-labial angle (°) | 5.6, (-36.3 to 18.7) | 0.072 | 0.099 |  | 0.015 | 0.451 |  | 0.003 | 0.751 |  | 0.015 | 0.451 |
| Lip-related indexes | |  |  |  |  |  |  |  |  |  |  |  |  |
|  | Upper vermilion height (mm) | 2.5, (7.4 to -2.2) | 0.001 | 0.823 |  | 0.001 | 0.870 |  | 0.003 | 0.753 |  | 0.001 | 0.870 |
|  | Lower vermilion height (mm) | 4.1, (9.2 to -0.3) | 0.074 | 0.094 |  | 0.022 | 0.364 |  | 0.052 | 0.163 |  | 0.022 | 0.364 |
|  | Vermilion height (mm) | 6.5, (14.0 to -0.1) | 0.039 | 0.230 |  | 0.012 | 0.499 |  | 0.031 | 0.285 |  | 0.012 | 0.499 |
| Bone-related indexes | |  |  |  |  |  |  |  |  |  |  |  |  |
|  | Morphological face length (mm) | 12.1, (34.5 to -5.3) | 0.033 | 0.270 |  | 0.040 | 0.225 |  | 0.056 | 0.145 |  | 0.040 | 0.225 |
|  | Face breadth (mm) | 12.0, (30.6 to -8.8) | 0.052 | 0.161 |  | 0.011 | 0.535 |  | 0.057 | 0.813 |  | 0.011 | 0.535 |
|  | Gonion-gnathion distance (mm) | 6.0, (23.3 to -14.4) | 0.122 | 0.029 |  | 0.011 | 0.522 |  | 0.002 | 0.782 |  | 0.011 | 0.522 |
| P value < 0.05 means the regression was significant. | | |  |  |  |  |  |  |  |  |  |  |  |
